# N-Acetylcysteine Mitigates Social Dysfunction in a Rat Model of Autism Normalizing Glutathione Imbalance and the Altered Expression of Genes Related to Synaptic Function in Specific Brain Areas

**DOI:** 10.3389/fpsyt.2022.851679

**Published:** 2022-02-25

**Authors:** Sara Schiavi, Piergiorgio La Rosa, Sara Petrillo, Emilia Carbone, Jessica D'Amico, Fiorella Piemonte, Viviana Trezza

**Affiliations:** ^1^Department of Science, University “Roma Tre”, Rome, Italy; ^2^Division of Neuroscience, Department of Psychology, Sapienza University, Rome, Italy; ^3^Neuromuscular and Neurodegenerative Diseases Unit, Bambino Gesù Children's Hospital, IRCCS, Rome, Italy

**Keywords:** N-acetylcysteine, valproic acid, rats, autism, glutathione

## Abstract

Prenatal exposure to valproic acid (VPA) is a risk factor for autism spectrum disorder (ASD) in humans and it induces autistic-like behaviors in rodents. Imbalances between GABAergic and glutamatergic neurotransmission and increased oxidative stress together with altered glutathione (GSH) metabolism have been hypothesized to play a role in both VPA-induced embriotoxicity and in human ASD. N-acetylcysteine (NAC) is an antioxidant precursor of glutathione and a modulator of glutamatergic neurotransmission that has been tested in ASD, although the clinical studies currently available provided controversial results. Here, we explored the effects of repeated NAC (150 mg/kg) administration on core autistic-like features and altered brain GSH metabolism in the VPA (500 mg/kg) rat model of ASD. Furthermore, we measured the mRNA expression of genes encoding for scaffolding and transcription regulation proteins, as well as the subunits of NMDA and AMPA receptors and metabotropic glutamate receptors mGLUR1 and mGLUR5 in brain areas that are relevant to ASD. NAC administration ameliorated the social deficit displayed by VPA-exposed rats in the three-chamber test, but not their stereotypic behavior in the hole board test. Furthermore, NAC normalized the altered GSH levels displayed by these animals in the hippocampus and nucleus accumbens, and it partially rescued the altered expression of post-synaptic terminal network genes found in VPA-exposed rats, such as *NR2a, MGLUR5, GLUR1*, and *GLUR2* in nucleus accumbens, and *CAMK2, NR1*, and *GLUR2* in cerebellum. These data indicate that NAC treatment selectively mitigates the social dysfunction displayed by VPA-exposed rats normalizing GSH imbalance and reestablishing the expression of genes related to synaptic function in a brain region-specific manner. Taken together, these data contribute to clarify the behavioral impact of NAC in ASD and the molecular mechanisms that underlie its effects.

## Introduction

Several factors such as gene mutations, gene variations and adverse environmental events concur to the pathogenesis of Autism Spectrum Disorder (ASD). Among the environmental factors involved in the pathogenesis of ASD, prenatal exposure to the antiepileptic drug valproic acid (VPA) has been repeatedly associated with increased risk of neurodevelopmental delay and autistic symptoms in the offspring (1, 2). When given during gestation, depending on the time window of exposure and the administered dose, VPA is able to cause congenital malformations (3, 4) and to induce core autistic symptoms in the offspring, such as impaired communication, reduced sociability and stereotyped behaviors (5, 6). Based on this clinical evidence, prenatal exposure to VPA in rodents has been validated as a preclinical model of ASD mimicking one of the environmental factors involved in the pathogenesis of the disease (7–9). Indeed, rodents exposed to VPA recapitulate core and comorbid features of ASD and are characterized by neurochemical alterations resembling those found in patients with autism, thus confirming the good face and construct validity of this preclinical model (7, 9–11).

Although the mechanisms by which VPA interferes with development causing autistic-like features in the exposed offspring need to be still elucidated, an imbalance between GABAergic and glutamatergic neurotransmission has been reported, which is also implicated in ASD etiology (12, 13). Indeed, defects in establishing and maintaining the balance between excitatory and inhibitory neurotransmission has been considered crucial in the pathogenesis of ASD (14). In particular, this imbalance might be responsible for disrupted neural connectivity and ASD-like behaviors such as impaired social interaction (15–19). Accordingly, male rats prenatally exposed to VPA show an excitatory/inhibitory imbalance, similar to ASD patients (20, 21). Moreover, the GABA excitatory-inhibitory shift that normally occurs during delivery is abolished in VPA-exposed animals, while restoring this shift rescues their aberrant behavioral phenotype (22).

Increased oxidative stress has been identified as a possible mechanism through which environmental factors exert their deleterious effects on the developing brain (23), which may be further exacerbated by their interaction with genetically susceptible alleles, thus increasing the risk of ASD. Not surprisingly, oxidative stress plays a role in the embriotoxicity induced by VPA (24, 25).

N-acetylcysteine (NAC) is a synthetic derivative of the endogenous amino acid L-cysteine that is able to modulate glutamatergic neurotransmission and the excitatory/inhibitory ratio (26, 27). Furthermore, the anti-oxidant properties of NAC are relevant to the pathogenesis of several neuropsychiatric disorders (28–33). Indeed, NAC is an essential substrate for the enzyme γ-glutamyl-cisteine synthetase (GCL) in the synthesis of glutathione (GSH), the main antioxidant in tissues (34). GSH redox imbalance plays an essential role in the onset and progression of several neurodegenerative and neuropsychiatric diseases and it may be used as a biomarker for their diagnosis (35). GSH deficit and GSH redox imbalance have been reported in individuals with autism, supporting a protective role of the GSH system in ASD development (36–41).

The antioxidant properties of NAC and its ability to modulate glutamatergic neurotransmitters have prompted studies focused on the potential clinical application of NAC in ASD (33). However, the studies conducted to date have provided controversial results, with some studies reporting beneficial effects of NAC administration in children with ASD (42), and other studies reporting minimal or no effects (41). In this context, basic research in validated preclinical models of ASD is essential to guide larger clinical trials in order to determine whether core symptoms of ASD may respond to treatment with NAC and to clarify the mechanisms involved in such effects.

Here, we explored the impact of repeated administration of NAC on core autistic-like features (such as social impairments and stereotyped behaviors) in the VPA rat model of ASD. Furthermore, since GSH redox imbalance seems to be specifically observed in some brain regions of autistic patients (37, 43), we measured GSH content in prefrontal cortex (PFC), dorsal striatum (DS), nucleus accumbens (NAc), amygdala (AMY), cerebellum (CER) and hippocampus (HIPP) of VPA-exposed animals, in order to understand if brain region-specific GSH variations may contribute to VPA-induced autistic-like traits. Last, the mRNA expression of genes encoding scaffolding (*PSD-95*), transcription regulation proteins (*MeCP2, CaMKII*), the subunits of NMDA (*NR1, NR2a*, and *NR2b*) and AMPA (*GLUR1* and *GLUR2*) receptors, as well as the metabotropic glutamate receptors *mGLUR1* and *mGLUR5*, that are all involved in the regulation of the post-synaptic terminal activity, was evaluated in the same brain regions.

## Materials and Methods

### Animals

**Figure 1:**
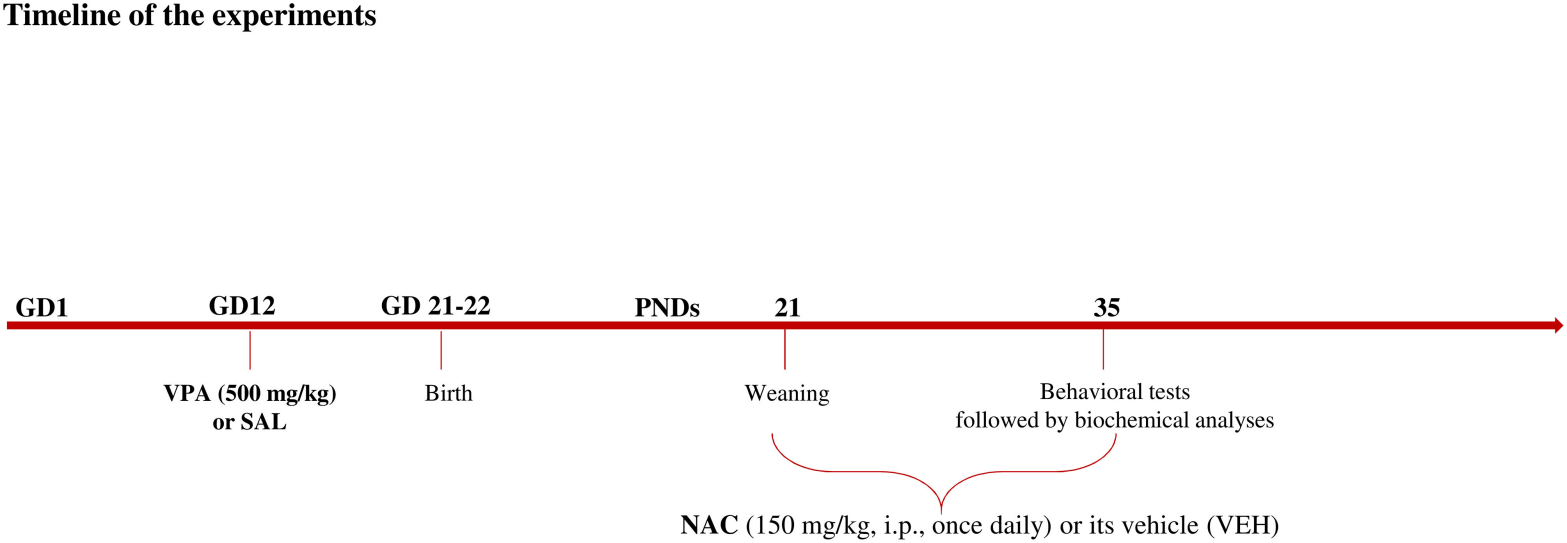
Timeline of the experiments.

Female Wistar rats (Charles River, Italy), weighing 250 ± 15 g, were mated overnight. The morning when spermatozoa were found was designated as gestational day 1 (GD 1). Pregnant rats were singly housed in Macrolon cages [40 (length) × 26 (width) × 20 (height) cm], under controlled conditions (temperature 20–21°C, 55–65% relative humidity and 12/12 h light cycle with lights on at 07:00 h). Food and water were available *ad libitum*. On gestational day 12.5, females received a single intraperitoneal (i.p) injection of either valproic acid (VPA) or saline (SAL; control group). Newborn litters found up to 17:00 h were considered to be born on that day [postnatal day (PND) 0]. On PND 1, the litters were culled to eight animals (six males and two females), to reduce the litter size-induced variability in the growth and development of pups during the postnatal period. On PND 21, the pups were weaned and housed in groups of three. The experiments were carried out on the male offspring during adolescence (PND 35) (see Figure 1). Different groups of animals belonging to the same cohort were utilized in the behavioral tests. Thus, the hole board and the three-chamber tests occurred at the same time in different experimental rooms using different animals. Right after the behavioral tests, the animals tested in the three-chamber test were sacrificed for biochemical analyses. One male pup per litter from different litters per treatment group was used in each experiment. The exact sample size (n) for each experimental group/condition is indicated in the figure legends. Sample size was based on our previous experiments and power analysis performed with the software GPower. Potential outliers within each data set were calculated using the GraphPad software using the Grubbs' method. A trained observer who was unaware of the treatments scored the behavioral tests using the Observer 3.0 software (Noldus, The Netherlands). The experiments were approved by the Italian Ministry of Health (Rome, Italy) and performed in agreement with the ARRIVE (Animals in Research: Reporting *in vivo* Experiments) guideline (44), the guidelines of the Italian Ministry of Health (D.L. 26/14) and the European Community Directive 2010/63/EU.

### Drugs and Treatment Schedule

VPA (Cayman Chemical, USA) was dissolved in saline at a concentration of 250 mg/ml and administered at a dose (500 mg/kg) and time (gestational day 12.5) that have been shown to induce autistic-like behavioral changes in the offspring (45, 46). Control dams were treated with saline solution (SAL). The offspring prenatally exposed to either VPA or SAL was treated with either N-acetylcysteine (NAC) (Sigma Aldrich, USA) or its vehicle (saline solution, VEH). NAC was administered to VPA- and SAL-exposed offspring at the dose of 150 mg/kg [intraperitoneally (i.p.)] daily for 15 days (47). Thus, four experimental groups were used:

1. Rats prenatally exposed to SAL and treated with VEH from PND 21 to PND 35 (Group SAL/VEH);
2. Rats prenatally exposed to VPA and treated with VEH from PND 21 to PND 35 (Group VPA/VEH);
3. Rats prenatally exposed to SAL and treated with NAC from PND 21 to PND 35 (Group SAL/NAC);
4. Rats prenatally exposed to VPA and treated with NAC from PND 21 to PND 35 (Group VPA/NAC).

The last NAC administration was given 2.5 h prior the behavioral experiments, as it has been demonstrated that glutamate levels were elevated in the rat brain approximately 2.5 h after NAC administration (48). Solutions were administered in a volume of 2 ml/kg.

### Behavioral Experiments

#### Three-Chamber Test

The test was performed as previously described (46). The apparatus was a rectangular three-chamber box, with two lateral chambers (30 × 35 × 35 cm; l × w × h) connected to a central chamber (15 × 35 × 35 cm; l × w × h). Each lateral chamber contained a small Plexiglas cylindrical cage. At PND 35, each experimental rat was individually allowed to explore the three-chamber apparatus for 10 min and then confined in the central compartment. An unfamiliar stimulus animal was confined in a cage located in one chamber of the apparatus, while the cage in the other chamber was left empty. Both doors to the side chambers were then opened, allowing the experimental animal to explore the apparatus for 10 min. The percentage of time spent in social approach (sniffing the stimulus animal) and the percentage of time spent exploring the empty chamber were scored using the Observer 3.0 software (Noldus, The Netherlands).

#### Hole Board Test

The test was performed in a sound attenuated chamber under dim light conditions, as previously described (45, 46). The apparatus consisted of a gray square metal table (40 l × 40 w × 10 h cm) with 16 evenly spaced holes (4 cm in diameter), inserted in a Plexiglas arena (40 l × 40 w × 60 h cm). Each rat was individually placed in the apparatus for 5 min. Each session was recorded with a camera positioned above the apparatus for subsequent behavioral analysis performed using the Observer 3.0 software (Noldus Information Technology, The Netherlands). Dipping behavior was scored by the number of times an animal inserted its head into a hole at least up to the eye level.

### Brain Sample Collection

Rats were rapidly decapitated, their brains were removed and cut into coronal slices on a cold plate. The PFC, DS, NAc, AMY, CER and HIPP were dissected by hand under microscopic control within 2 min as previously described (49–51).

### Glutathione Assay

Glutathione levels were detected in brain areas by an enzymatic re-cycling assay. Samples were de-proteinized with 5% (w/v) sulphosalycilic acid (SSA, Sigma-Aldrich, USA) and the glutathione content was determined after dilution of the acid soluble fraction in Na-phosphate buffer containing EDTA (pH 7.5). Free GSH and Total GSH concentrations were measured with the ThioStar® glutathione detection reagent (Arbor Assays, USA), using GSH as standard (Sigma Chemicals, USA), and expressed as nmol/mg proteins. After 15 min reaction, Free GSH was read, followed by the addition of a reducing mixture that converts all the oxidized glutathione thus allowing the measurement of Total GSH. The fluorescence intensity was measured by an EnSpire® Multimode Plate Reader (Perkin Elmer, USA).

### RNA Extraction and qRT-PCR

Total RNA from brain tissues was extracted as reported (52). After quantification, 1 μg of RNA was retro-transcribed by M-MLV reverse transcriptase (Invitrogen, CA, United States) and used in quantitative RT-PCR (qPCR) experiments using Power-up Sybr green PCR master mix (Applied Biosystem, CA, United States) following manufacturer's instructions. Primers used are listed in Table 1. L34 gene expression was used to normalize qPCR experiments.

**Table 1:** List of oligonucleotides pairs used for qPCR analysis in this study.

**Gene**	**Primer sequence**
mGlur1	FW: AAAATTCGCAGCCAACAGGG
	RV: CCAGCCGACTCCGTTCTAAG
NR1	FW: AAACCTCGACCAACTGTCCT
	RV: ATCAGCAGAGCCGTCACATT
NR2A	FW: GAACCCGCTAAACCTGGTGG
	RV: CATGGTCGCCACTTAGGGTC
NR2B	FW: GTCCTTCAGCGAAGATGGCT
	RV: CACATCCGAGGCCACACATA
mGluR5	FW: AAACCCTAAGCTCCAACGGAA
	RV: GAAAGGGTTTGATGACCGCC
GLUR1	FW: GGACAACTCAAGCGTCCAGA
	RV: CACAGTAGCCCTCATAGCGG
GLUR2	FW: GCATCGCCACACCTAAAGGA
	RV: TACTTCCCGAGTCCTTGGCT
PSD95	FW: CCGCTACCAAGATGAAGACACG
	RV: TCTGTTCCATTCACCTGCAACTC
CAMKII	FW: AGACACCAAAGTGCGCAAAC
	RV: TTCCAGGGTCGCACATCTTC
MeCP2	FW: AGAGGAAGTCTGGTCGCTCT
	RV: AGGAGGTGTCTCCCACCTTT

### Statistical Analysis

Behavioral data are expressed as mean ± S.E.M. Two-way analysis of variance (ANOVA) was used to assess the effects of prenatal and postnatal treatments in the three-chambers and hole board tests, using prenatal (VPA or SAL) and postnatal [NAC or vehicle (VEH)] treatments as between-subjects factor. The results of the biochemical experiments (GSH content and qPCR) are expressed as mean ± SD. GSH content data were analyzed by One-way ANOVA while qPCR data were analyzed by Two-way ANOVA. One- and Two-way ANOVAs were followed by either Student's-Newman-Keuls (for behavioral data) or Tukey's multiple comparison (for biochemical data) post hoc tests where appropriate. The software GraphPad Prism 8 (GraphPad Software, USA) was used to perform the statistical analysis of both biochemical and behavioral experiments. All biochemical experiments were performed in triplicates. The accepted value for significance was set at *p* < 0.05.

## Results

### N-Acetylcysteine Mitigates the Aberrant Social Phenotype but It Does Not Affect the Repetitive Behaviors Displayed by VPA-Exposed Animals

**Figure 2:**
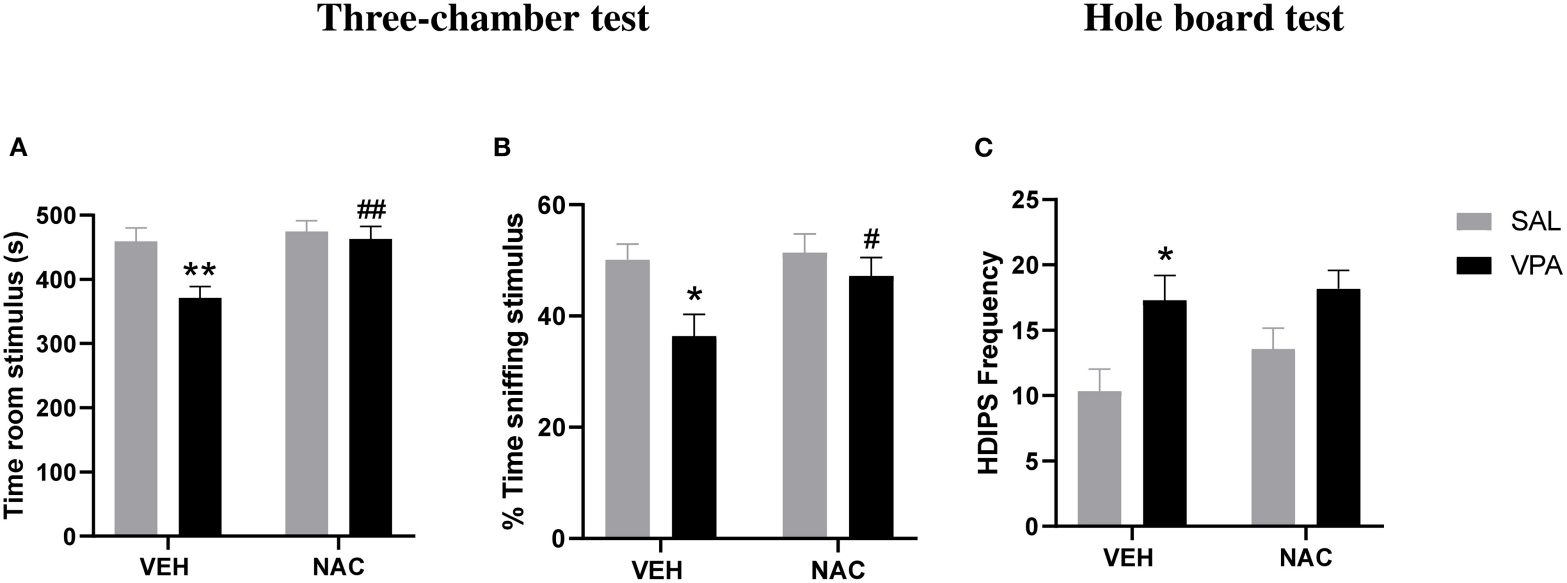
N-acetylcysteine ameliorates the aberrant social phenotype but it does not affect the repetitive behaviors displayed by VPA-exposed animals. At PND 35, VPA-exposed rats showed reduced sociability in the three-chamber test, as they spent less time in the stimulus room **(A)** and sniffing the stimulus animal **(B)** compared to SAL-exposed rats. Treatment with NAC reversed the deficit displayed by VPA-exposed rats in this test (SAL-VEH, *n* = 7; SAL-NAC, *n* = 8; VPA-VEH, *n* = 6; VPA-NAC, *n* = 8). Moreover, VPA-exposed rats showed stereotypic behaviors in the hole-board test, as they made more head dippings **(C)** compared to SAL-exposed rats. Treatment with NAC was not able to rescue the repetitive behavior found in VPA-exposed rats (SAL-VEH, *n* = 6; SAL-NAC, *n* = 7; VPA-VEH, *n* = 10; VPA-NAC, *n* = 12). Data represent mean ± S.E.M. *p < 0.05, **p < 0.01 vs. SAL-VEH group; #*p* < 0.05, ##*p* < 0.01 vs. VPA-VEH group (Student's–Newman–Keuls *post hoc* test).

At PND 35, VPA and SAL-exposed animals treated with NAC or its vehicle (VEH) were tested in the three-chamber test. A two-way ANOVA analysis performed on the percentage of time spent sniffing the stimulus animal and the time spent in the stimulus room gave the following results: *time spent in the stimulus room* [*F*_(prenatal treat.)(1,25)_ = 6.875, *p* < 0.05.; *F*_(postnatal treat.)(1,25)_ = 7.997, *p* < 0.01; *F*_(prenatal treat. × postnatal treat.)(1,25)_ = 4.065, *p* = n.s.]; *% time spent sniffing stimulus* [*F*_(prenatal treat.)(1,25)_ = 6.895, *p* < 0.05.; *F*_(postnatal treat.)(1,25)_ = 3.117, *p* = n.s; *F*_(prenatal treat. × postnatal treat.)(1,25)_ = 1.951, *p* = n.s.]. *Post hoc* analysis revealed that VPA-exposed rats spent less time in the stimulus room (^**^p < 0.01 vs. rats prenatally exposed to SAL, Figure 2A) and sniffing the stimulus animal (^*^p < 0.05 vs. rats prenatally exposed to SAL, Figure 2B) compared to SAL-exposed rats, indicating reduced sociability. Treatment with NAC normalized the deficit displayed by VPA-exposed rats in this test (*time spent in the stimulus room*, ^##^*p* < 0.01 vs. VPA-exposed rats treated with VEH; *% time spent sniffing stimulus*, ^#^*p* < 0.05 vs. VPA-exposed rats treated with VEH).

To exclude that NAC treatment somehow impacted on locomotor activity, we also scored the number of crossing made by each experimental animal between each of the three compartments of the three-chamber apparatus. A two way ANOVA analysis of this parameter gave the following results: *F*_(pretreatment)(1,25)_ = 0.043, *p* = n.s.; *F*_(postnatal treat.)(1,25)_ = 3.602, *p* = n.s.; *F*_(prenatal treat. × postnatal treat)(1,25)_ = 0.069, *p* = n.s. (data not shown), revealing no locomotor impairment or possible sedative effects induced by NAC at the tested dose.

Moreover, VPA-exposed rats showed stereotypic behaviors in the hole-board test, as they made more head dippings [*F*_(prenatal treat.)(1,31)_ = 10.80, *p* < 0.01.; *F*_(postnatal treat.)(1,31)_] = 1.361, *p* = n.s; *F*_(prenatal treat. × postnatal treat.)(1,31)_ = 0.454, *p* = n.s] compared to SAL-exposed rats (^*^p < 0.05 vs. rats prenatally exposed to SAL, Figure 2C). Treatment with NAC was not able to reduce the stereotypic behavior found in VPA-exposed rats.

### N-Acetylcysteine Normalizes the Altered Brain GSH Levels Displayed by VPA-Exposed Animals in a Region-Specific Manner

**Figure 3:**
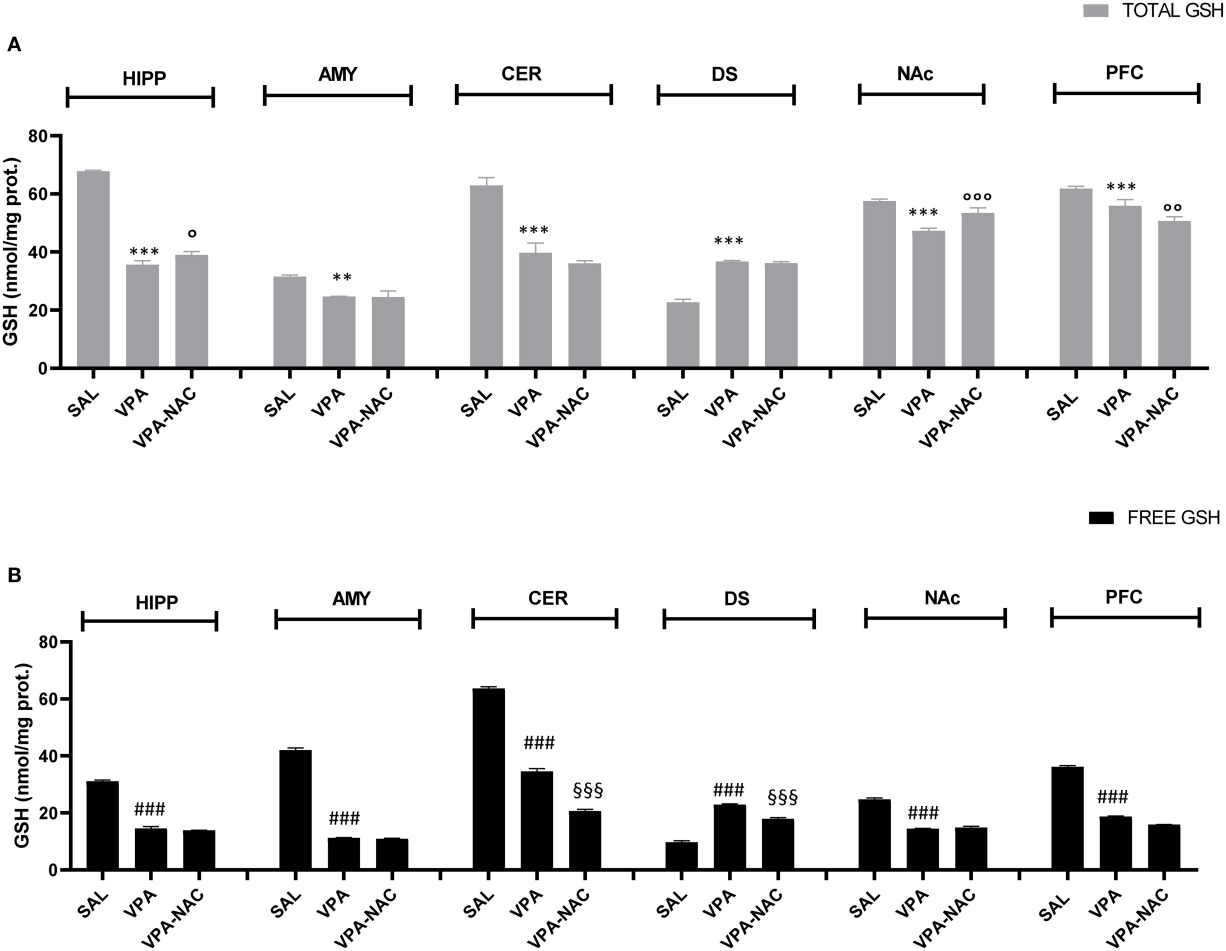
Brain region specific changes in total **(A)** and free **(B)** GSH levels. Total **(A)** and free **(B)** GSH levels in hippocampus (HIPP), amygdala (AMY), cerebellum (CER), dorsal striatum (DS), nucleus accumbens (NAc), and prefrontal cortex (PFC) of VPA-, SAL- and VPA-NAC-treated rats (*n* = 3 per group). Data represent mean ± SD. **p < 0.01, ***p < 0.001, vs. SAL-group TOTAL GSH; ^###^*p* < 0.01 vs. SAL-group FREE GSH; °*p* < 0.05, °°*p* < 0.01, °°°*p* < 0.001 vs. VPA-group TOTAL GSH; ^§§§^*p* < 0.001 vs. VPA-group FREE GSH (Tukey *post-hoc* test).

GSH levels were measured in different brain areas of VPA-exposed animals and SAL-exposed controls, in order to verify if brain region-specific changes in GSH occur in this animal model of ASD. As shown in Figures 3A,B, GSH concentration was significantly decreased in most brain areas of VPA-exposed animals (HIPP, AMY, CER, NAc, PFC) compared to SAL-exposed controls, either as total GSH amount [HIPP: *F*_(2,6)_ = 941.2, *p* < 0.001; AMY: *F*_(2,6)_ = 32.03, *p* < 0.001; CER: *F*_(2,6)_ = 96.39, *p* < 0.001; NAc: *F*_(2,6)_ = 51.85, *p* < 0.001; PFC: *F*_(2,6)_ = 38.42, *p* < 0.001, Figure 3A], or as GSH free form (not protein bound) [HIPP: *F*_(2,6)_ = 1,440, *p* < 0.001; AMY: *F*_(2,6)_ = 3,906, *p* < 0.001; CER: *F*_(2,6)_ = 2,438, *p* < 0.001; NAc: *F*_(2,6)_ = 736.3, *p* < 0.001; PFC, *F*_(2,6)_ = 3,681, *p* < 0.001, Figure 3B], except for DS where GSH content was higher in VPA-exposed rats than in controls [GSH total: *F*_(2,6)_ = 375.7, *p* < 0.001, Figure 3A; GSH free: *F*_(2,6)_ = 660.2, *p* < 0.001, Figure 3B]. Interestingly, when VPA-exposed rats were treated with NAC, only HIPP and NAc displayed a significant increase of total GSH concentrations. Overall, these findings support an imbalance of GSH homeostasis induced by prenatal VPA exposure and suggest a brain region specificity in the effects of NAC on GSH homeostasis.

### N-Acetylcysteine Partially Rescues the Altered Expression of Post-synaptic Terminal Network Genes Found in VPA-Exposed Rats

**Figure 4:**
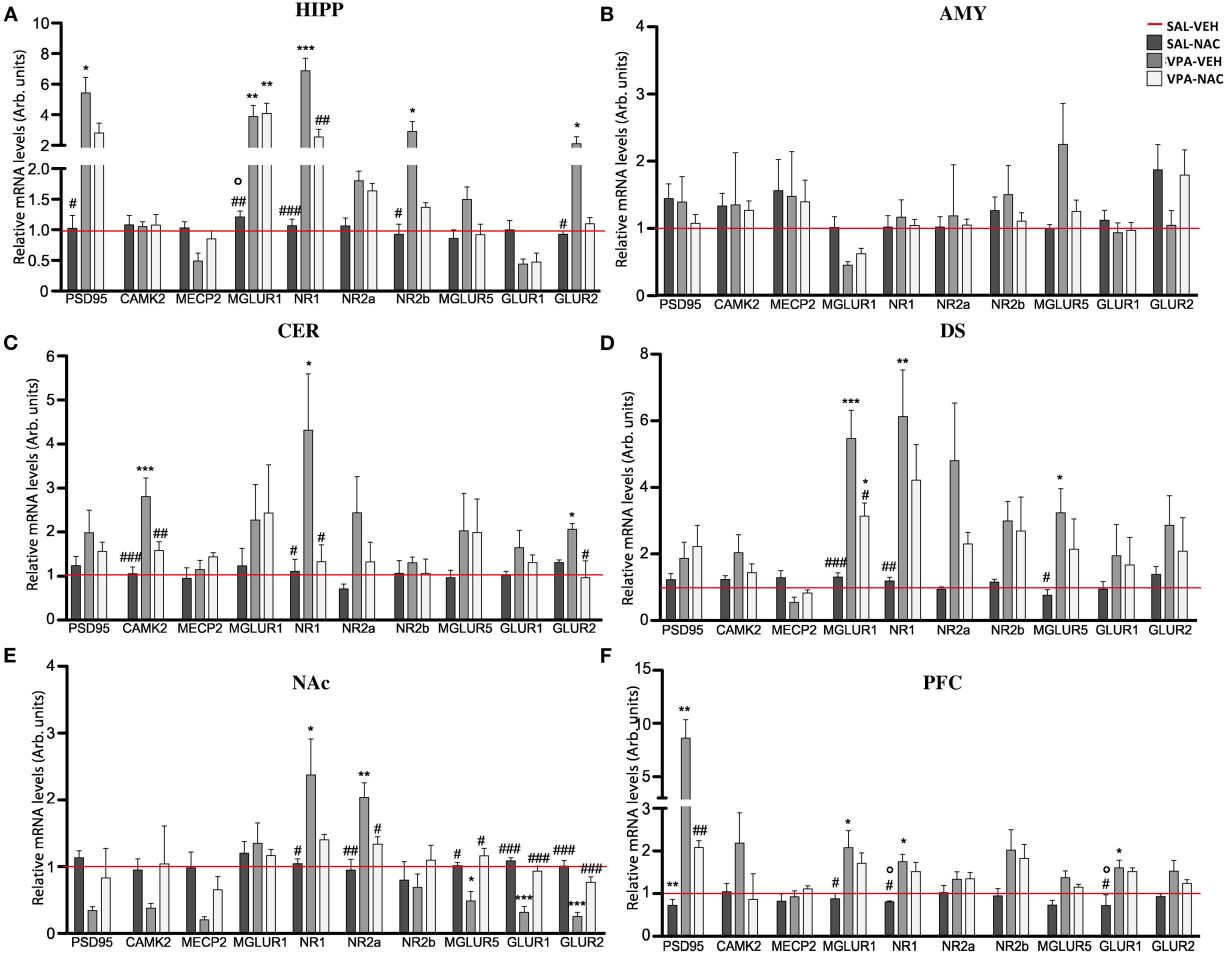
N-acetylcysteine partially rescues the altered expression of post-synaptic terminal network genes found in VPA-exposed rats. Relative mRNA levels of scaffolding (*PSD95*) and transcriptional regulators (*CAMK2* and *MeCP2*), ionotropic NMDA receptor subunits (*NR1, NR2a*, and *NR2b*), ionotropic AMPA receptor subunits (*GLUR1* and *GLUR2*) and metabotropic glutamate receptors *MGLUR1* and *MGLUR5*, assessed in HIPP **(A)**, AMY **(B)**, CER **(C)**, DS **(D)**, NAc **(E)**, and PFC **(F)** of rats prenatally exposed to either VPA or SAL and treated with NAC or its vehicle (VEH) (*n* = 3–4). Values represent mean ± SD. *p < 0.05, **p < 0.01, ***p < 0.001 vs. SAL/VEH group; ^#^*p* < 0.05, ^##^*p* < 0.01, ^###^*p* < 0.001 vs. VPA/VEH group; °*p* < 0.05 vs. VPA/NAC group (Tukey *post-hoc* test).

We evaluated the mRNA expression of genes that regulate the neuronal post-synaptic terminal activity, including scaffolding (*PSD-95*) and gene transcription regulator (*MeCP2, CaMKII*) proteins, the subunits of the ionotropic N-methyl-D-aspartate (NMDA) (*NR1, NR2a*, and *NR2b*) and α-amino-3-hydroxy-5-methyl-4-isoxazolepropionic acid (AMPA) (*GLUR1* and *GLUR2*) receptors, as well as the metabotropic glutamate receptors *mGLUR1* and *mGLUR5*. Our analysis demonstrates that, with the exception of the AMY (Figure 4B), all of the brain areas analyzed of VPA-exposed rats showed a deregulation in the expression of the genes that mediate glutamate signaling in the post-synaptic terminal (Figures 4A,C–F). Although these impairments were region specific, a general upregulation of the analyzed transcripts was observed, with levels that, in case of the *PSD95* transcript in the PFC (Figure 4F) and HIPP (Figure 4A), reached 8 (*p* < 0.001) or 6 (*p* < 0.05) fold the control levels, respectively. A general upregulation of *MGLUR1* and NMDAR subunits was also observed along most of the brain areas of VPA-exposed animals, with peaks 5-fold higher than the control levels in *MGLUR1* (*p* < 0.001), *NR1* (*p* < 0.01), and *NR2a* (*p* < 0.05) transcripts observed in the DS (Figure 4D). Conversely, a general VPA-induced downregulation was observed in the NAc, particularly in the expression of the AMPA receptor subunits, with a 70% reduction of *GLUR1* (*p* < 0.001) and *GLUR2* (*p* < 0.01) and a 50% reduction of the metabotropic glutamate receptor *MGLUR5* (*p* < 0.05). The sole exceptions observed regard the expression of *NR1* and *NR2a* subunits of the MNDA receptor, which were upregulated (*p* < 0.05 and *p* < 0.01, respectively) in VPA-exposed rats (Figure 4E).

Noteworthy, a partial rescue in the expression of the post-synaptic terminal network genes was observed in VPA-exposed rats after NAC treatment, with a trend that was, even in this case, region specific. In particular, a strong re-establishment of the physiological mRNA expression of the post-synaptic terminal genes occurred in the NAc, where the expression of 4 out of the 10 transcripts analyzed was rescued (*NR2a, MGLUR5, GLUR1*, and *GLUR2*) (Figure 4E), and in the CER (Figure 4C), where CAMK2, NR1, and GLUR2 expression was reduced (*p* < 0.05). No effects were observed upon NAC exposure in control rats.

## Discussion

Although ASD is among the most severe chronic childhood disorders, it lacks effective treatments, and its complex etiology is still a matter of investigation. Multiple factors are thought to be involved in the pathogenesis of the disease (i.e., genetic, environmental factors and their interaction), thus explaining the heterogeneity of symptoms displayed by autistic patients.

In recent years, several studies from different research groups reported reduced GSH levels in the brain and blood of ASD children (36, 38, 53). This depletion of GSH may be critically involved in the pathogenesis of ASD through different mechanisms. First, it may lead to increased oxidative stress toxicity. Second, it may induce excitotoxicity through over-activation of excitatory glutamatergic neurotransmission (54). Third, as GSH is an epigenetic modulator, its deficiency may negatively affect brain development in the presence of triggering environmental factors, thus contributing to ASD. One of the environmental factors known to be involved in the pathogenesis of ASD is maternal exposure to VPA. There is indeed clinical evidence that maternal use of VPA during pregnancy can induce a wide range of abnormalities in the exposed children, ranging from structural malformations to more subtle autistic-like behaviors (1, 5, 8, 55, 56). Based on the robust clinical evidence, prenatal exposure to VPA in rodents has been validated as an environmentally-triggered preclinical model of ASD (8, 9, 57).

Since NAC has antioxidant properties, it is a precursor of glutathione and it modulates glutamatergic neurotransmission, we explored the effects of repeated NAC administration on core autistic-like features, brain GSH metabolism and mRNA expression of genes that regulate synaptic activity in the VPA rat model of ASD.

We found that repeated administration of NAC reverted the social deficit displayed by VPA-exposed rats in the three-chamber test. Since the last NAC administration was given 2.5 h prior the behavioral experiments, we cannot exclude that the animals were still under acute NAC influence when performing the task. Yet, repeated administration of NAC has previously found to be effective: when NAC was administered for 10 days at the same dose used in the present study, it reverted the deficit in social interaction displayed by male VPA-exposed rats (27). However, while NAC was found to ameliorate the repetitive behaviors performed by VPA-exposed rats in the open field test (47), we found that NAC did not counteract the repetitive/stereotyped behaviors displayed by VPA-exposed rats in the hole board test. Thus, the positive effect of NAC on stereotypic behaviors in VPA-exposed animals may be dependent on the behavioral task and the experimental protocol used. To support the preclinical findings, a recent meta-analysis of randomized clinical trials showed that NAC supplementation for 8–12 weeks significantly improved irritability, hyperactivity and social awareness in ASD patients (58), thus supporting the idea that certain behavioral traits observed in autism may benefit of NAC treatment.

The brain is particularly susceptible to oxidative stress, due to high oxygen consumption (~20% of oxygen), low endogenous antioxidant defense, large quantity of iron and copper, and abundance of polyunsaturated fatty acids in neuronal membranes (59).

GSH plays a key role in the maintenance of the brain redox equilibrium and imbalances in the GSH redox system are an important factor in the pathophysiology of ASD (36, 38, 53, 60).

Some studies have previously reported significantly reduced GSH levels in *post mortem* cerebellum and temporal cortex of children with ASD, along with lower activity of the enzyme responsible for the GSH synthesis, γ-glutamyl cysteine ligase (GCL) (35, 37, 40, 60). In light of this, we measured brain GSH levels in VPA-exposed rats, also exploring the potential region-specificity of these changes. Our findings show that, compared to SAL-exposed rats, GSH concentrations are significantly decreased in the prefrontal cortex, nucleus accumbens, amygdala, cerebellum and hippocampus of VPA-exposed animals. These data support the GSH depletion found in *post mortem* cerebellum of children with ASD but, at the same time, they highlight a more generalized GSH deficiency in several brain regions, with the only exception for the dorsal striatum, where GSH levels appear increased.

Two principal factors can be responsible for the reduction of GSH levels found in VPA-exposed animals: a decreased synthesis, due to a direct or indirect inhibition of the enzyme GCL, and/or an increase of the GSH consumption by reactions catalyzed by the GSH-dependent antioxidant enzymes (GPx, GR, GST). Exposure to toxic metals, for instance, can block GSH synthesis and rise thiol excretion, thus exacerbating redox dysfunctions (61, 62). Also the gut microbiota, which is dysregulated in ASD, has been found to modulate glutathione metabolism (63), potentially contributing to predispose children to the gastrointestinal dysfunction often occurring in the autistic phenotype (38, 64–66).

In addition, and besides its well-known role as antioxidant, GSH can affect “redox-independent” processes, including the accumulation of extracellular glutamate, thus triggering excitotoxicity and ultimately resulting in neuronal dysfunction and death (36, 67, 68).

GSH metabolism is strongly inter-connected with glutamatergic neurotransmission: the exposure to glutamate results in a dose-dependent decrease of GSH in astrocytes (69) but, at the same time, it represents an essential precursor of the GSH synthesis (70). Furthermore, GSH elicits a modulatory function on glutamate receptors (such as N-methyl-D-aspartate receptors) and regulates some important transcription factors implicated in the immune response pathway (NF-kB) and in neuro-inflammation. Therefore, the GSH homeostasis is crucial for neuronal health and neurodevelopment contributing to the balanced interaction between glutamate and GABA, both essential for synaptic maturation, refinement of neuronal circuitry and regulation of cognition, emotion and behavior (54, 71–73).

Moving from previous studies showing that VPA-induced behavioral deficits in rodents can be rescued by NAC treatment (27, 47), through the GSH-mediated modulation of synaptic activity, here we analyzed the effect of NAC on GSH brain levels in VPA-exposed rats. Unexpectedly, total and free GSH levels did not show significant variations after NAC supplementation, except for two brain regions, the nucleus accumbens and hippocampus, where total GSH (an index of GSH synthesis) was consistently increased. The reason of this differential, area-specific effectiveness of NAC on GSH levels is not still understood, but it has to be considered that NAC is involved not only in GSH production, but in multiple processes underlying the pathophysiology of several neuropsychiatric disorders (74). Indeed, beside its essential role as reducing agent (by its thiol group) and as co-substrate for the GSH synthesis, NAC has also anti-glutamatergic properties, and NAC-treated mice showed reduced amounts of glutamate and excitatory currents *in vivo* (74–77). Thus, the ability of NAC to rescue the social deficits displayed by VPA-exposed rat, rather than derive from a direct increase in GSH levels, can be the result of its central role in glutamate signaling, oxidative homeostasis and interaction with inflammatory mediators.

An imbalance of the excitatory synaptic signaling is widely reported in ASD (78, 79), especially regarding the excess of glutamatergic signaling (80) connected to impairments in the synaptic proteins expression at the synaptic terminals (particularly in males) (16, 20, 21, 81). For this reason, we evaluated the mRNA expression of genes regulating the neuronal post-synaptic terminal activity in VPA-exposed animals and SAL-exposed controls. Our data confirm that prenatal VPA exposure induces a general region-specific impairment in the expression of genes related with synaptic regulation, and that NAC treatment is able to partially rescue these defects to normal levels or, at least, to reduce their impact. On this basis, it is possible to hypothesize a role for NAC in re-establishing the physiological glutamatergic signaling, which appears altered in ASD. Taken together our results corroborate previous findings (27, 47) showing that NAC administration improves VPA-induced behavioral deficits in rodents, such as anxiety, social impairments and stereotypic behavior, modulating synaptic activity through its antioxidant effects and its ability to modulate glutamatergic neurotransmission.

Overall, this study highlights a general brain depletion of GSH in the VPA rat model of ASD and, interestingly, suggests a differential response of different brain areas to the antioxidant treatment with NAC. However, what still needs to be determined is if the GSH decrease represents a primary defect in ASD or if the ASD pathological assessment leads to a GSH redox imbalance. Nevertheless, it is certain that the GSH deficiency results in a decreased ability to excrete toxic molecules (metals, xenobiotics) that, instead, an antioxidant supplementation may help to counteract.

## Data Availability Statement

The raw data supporting the conclusions of this article will be made available by the authors, without undue reservation.

## Ethics Statement

The animal study was reviewed and approved by Italian Ministry of Health Autorizzazione n. 70/2021-PR.

## Author Contributions

SS performed, analyzed, and contributed to the design of the behavioral experiments. SP and PL performed, analyzed, and designed the biochemical experiments. EC and JD'A contributed to the behavioral and the biochemical experiments, respectively. SS, PL, and SP wrote the manuscript. VT and FP supervised the project, designed the experiments, and wrote, revised, and edited the manuscript. All authors contributed to the article and approved the submitted version.

## Funding

VT was supported by Jerome Lejeune Foundation Research grant #1674 (VT), by PRIN 2017 grant (2017SXEXT5) by MIUR (VT), and by Excellence Departments, MIUR-Italy, Grant/Award Numbers: ARTICOLO 1, COMMI 314-337 LEGGE 232/2016, ARTICOLO 1.

## Conflict of Interest

The authors declare that the research was conducted in the absence of any commercial or financial relationships that could be construed as a potential conflict of interest.

## Publisher's Note

All claims expressed in this article are solely those of the authors and do not necessarily represent those of their affiliated organizations, or those of the publisher, the editors and the reviewers. Any product that may be evaluated in this article, or claim that may be made by its manufacturer, is not guaranteed or endorsed by the publisher.
